# Lipid composition and nutritional quality of some commercially available cold pressed oils

**DOI:** 10.3389/fnut.2025.1721761

**Published:** 2025-12-18

**Authors:** Lisa L. Dean

**Affiliations:** Food Science and Market Quality and Handling Research Unit, USDA, Agriculture Research Service, Raleigh, NC, United States

**Keywords:** unrefined oil, benne, okra, peanut, pecan, pumpkin, sunflower, oil quality

## Abstract

Consumer interest in alternatives to highly refined oils from soybean and corn for culinary applications has resulted in an increase in the availability of alternatives, especially cold pressed ones. Sources that were once only known to certain regions or cultures are now becoming more mainstream. In addition, the interest in sustainability in the agricultural sector has led to the usage of seeds from previous “waste” sources. The fatty acid profiles, phytosterols and tocopherols were evaluated in some cold pressed oils from small processors. These included benne (black sesame), okra seeds, peanuts, pecans, pumpkin seeds, and sunflower seeds. Polyunsaturated fatty acid contents ranged from 1.8% in sunflower oil to 44.3% in pumpkin seed oil. Cold pressed oils do not have the phytosterols and tocopherols removed by further refining. All the oils tested contained significant amounts of phytosterols with the lowest levels of Beta-sitosterol in the okra seed oil (29.0 mg/100 g) and the highest in the sunflower seed oil (251 mg/100 g). The tocopherols present in the oils were in agreement with literature reports for oil seeds and tree nuts with significant amounts of the alpha and gamma forms. Unusually, the benne oil was found to have large amounts of the Beta form (8.8 mg/g oil). Use of these oils can make positive contributions to human health by providing significant amounts of these lipid nutrients to the diet.

## 1 Introduction

Oils of vegetable origins are the primary sources of lipids as human food. Edible oils are produced from a wide variety of grains, plants and especially seeds. The greatest portion is produced commercially from a few sources requiring almost one fourth of the world’s cropland to meet the demand (1). These major sources include soybean, rapeseed, palm and sunflower with lesser amounts from olive, coconut, corn (maize), peanut and cottonseed (2). While vegetable oils are used as ingredients in a wide range of processed food products and in cosmetic and pharmaceutical applications, the major use is as a heat transfer medium in the cooking of foods, especially deep frying (3). On the industrial scale, their production involves recovery of the oil by extraction with organic solvents such as hexane, or to a lesser extent by crushing or pressing (4). The recovered oils are then further processed to remove minor lipid components that are considered undesirable due to their interference in certain functions of edible oils or for their effects on physical properties.

In addition to the avoidance of possible contamination by extraction solvents, the cold pressing of oils has the advantage of minimal losses of various compounds naturally present in the oil. While the oil is primarily triglycerides, other compounds such as phytosterols, phospholipids, glycolipids, mono and diglycerides are present in much smaller quantities. These minor components are known to have positive health effects (5). Maintaining them in oils for consumers would be beneficial. Small volume specialty culinary oil production makes use of a range of seed sources resulting in oils which differ from bulk refined cooking oils. The cold pressed oils are often highly colored, with distinct flavors and become cloudy at low temperatures (6). They are often more easily oxidized resulting in shorter shelf lives, low smoke points, and are generally not suited to high temperature cooking operations such as deep frying or oil roasting (7). Their flavor profiles also make them unsuitable as ingredients where the functionality of the oil also requires a bland flavor such as baked goods.

### 1.1 Sources of cold pressed oils

#### 1.1.1 Benne

Seeds of the sesame plant (*Sesamum indicum* L.) were one of the earliest sources of oil for human consumption (8). Although the white varieties are more widely cultivated for sesame oil, the black seeds are also a source of food oils, especially in Africa and the oil is known as Benne. The older literature showed that black seeds had lower levels of saturated fatty acids than the white seed which could lead to greater stability to oxidation (9). The resulting oil has a rather complex flavor that lends itself to applications such as salad dressings.

#### 1.1.2 Green peanut

The peanut plant (*Arachis hypogaea* L.) is unique in that it is a legume which produces its seed in woody pods underground. At harvest, the plants are pulled loose from the soil and turned up to allow the seed pods to air dry. The pods are then mechanically removed from the rest of the vegetative plant matter for further processing. For long term storage, the pods are further dried. To produce products such as green peanut for boiling, the seeds are immediately processed without further drying. Further drying to prevent mold growth upon storage is done when the pods arrive at storage warehouses (10). The dried peanuts are shelled to produce peanuts for oil. Cold pressed oil from green peanut is darker in color than refined peanut oil and maintains a “green” aroma due to the presence of certain aldehydes and flavor like a fresh pea (6). The aroma is not as intense as some further processed oils such as those from roasted peanuts due to lower levels of volatile compounds being produced (11).

#### 1.1.3 Okra seed

Okra (*Abelmoschus esculentus* L.) is a perennial plant that produces fruit in the form of a finger shaped capsule that contains numerous seeds. It is produced globally in tropical and semitropical regions where there is no danger of frost. In the United States, it has been incorporated into the cuisines of the southeastern regions for over 300 years (12). Although it is grown to produce pods for use as food, the seeds contain significant amounts of oil that has proven suitable for food use (13). As the okra plant is related to cotton, the oil does contain gossypol, an antinutritional metabolite of sesquiterpene that is potentially toxic, but the levels have been found to be less than 10% of the recommended FDA maximum (14). The oil has been noted to have a distinct vegetable “spice” like flavor (15). This flavor does not appear to interfere with the use of the oil as a bakery ingredient (16), but presently availability and cost limit its use to that of a table dressing.

#### 1.1.4 Pecan

Commonly consumed tree nuts are noted for their high lipid contents which can range from 43% in cashews to over 75% in macadamia nuts (17). This makes them excellent sources of edible oils. The pecan [*Carya illinoinensis* (Wangenh.) K. Koch] is usually classified as a tree nut, but like the cashew is actually a drupe with the nut being the seed. The lipid level is high at 72% and pecans are noted for their sweet “buttery” type flavor. They are native to the United States produced mostly in the Southern and western portions and in Mexico with minor production in South Africa, Brazil and several other countries across the world (18). With such a high lipid content, they are an excellent source of cold pressed oil, although yields are reported to be low at only 58% (19). It has a distinct “nutty” aroma and flavor that consumers find appealing for use as a salad oil, but may limit its applications as a cooking oil (20).

#### 1.1.5 Pumpkin seed

The seeds of certain members of the gourd or squash family (*Cucurbitaceae*) are excellent sources of vegetable oils. In particular, pumpkins (*Cucurbitaceae pepo*) which are grown in many areas of the world for their flesh as a source of food, their seeds for oil and for snack food, all parts as livestock feed and in general as a decorative item. In the United States, they are grown in most states where the growing season is hot with and not overly wet. They need to be harvested before there is a danger of frost (21). Pumpkin seeds have lipid contents of 35 to 50%, making them a significant source of oil (22). The oil is darker in color than most vegetable oils and the flavor is described as intensely nutty and sometimes slightly bitter (23). It is considered a good choice as a dressing oil and for marinades, but not for high temperature applications such as cooking due to the oil being very polyunsaturated. In addition to food uses, pumpkin seed oil has been noted globally for a range of uses such as treatment of benign prostatic hyperplasia, prevention of hair loss, cardiovascular protective effects, antiulcer agent, a sunscreen ingredient, and a facial cleanser among others (24).

#### 1.1.6 Sunflower seed

Of the various seed sources for cold pressed oils discussed here, oil from the seeds of the sunflower (*Helianthus annuus* L) is the most widely available. The seeds are produced commercially on a large scale for cooking oil worldwide. Sunflower ranks third among oil seeds behind only soy and rapeseed (25). Sunflower seeds have an oil content of 40 to 50% making them an excellent candidate for cold pressing (26). The oil is noted for its neutral flavor, making it suitable for most food purposes and commercially it is often used for producing margarines and as a baking ingredient (27). High oleic varieties have been developed which give the oil more stability against oxidation which has enhanced its usefulness both in food and nutraceutical purposes.

## 2 Methods

### 2.1 Research materials

Retail bottles of Benne, Green Peanut, Okra Seed, Pecan, Pumpkin Seed, and Sunflower Seed Oil were obtained from Oliver Artesian Oils (Pitts, GA, United States) for analysis. All chemicals and reagents used were obtained from Thermo Fisher Inc. (Waltham, MA, United States) unless otherwise noted.

### 2.2 Fatty acid profiles

The fatty acid profiles of the oils were determined as previously described (28). In brief, the oils were directly saponified with methanolic sodium hydroxide and the released fatty acids were methylated using boron trifluoride (14% in methanol, Sigma Aldrich, St. Louis, MO, United States) as the catalyst. The methyl esters were extracted into hexane. The extracts were analyzed by gas chromatography with flame ionization detection using a Perkin Elmer XL Autosampler system (Shelton, CT, United States). The column was a BPX-70 (30 m length, 0.25 mm interior diameter, 0.25-micron film, Phenomenex Corp., Torrance, CA, United States). The temperature program was 60 °C held for 2 min, then increased at 10 °C/min to 180 °C, then increased at 4 °C/min to 235 °C. The carrier gas was helium at 2.5 mL/min. The injector was split at 50 mL/min with a temperature of 220° and detector temperature was 235 °C. A standard mix of methyl esters (Kel Fir Fame 5, Matreya LLC, Pleasant Gap, PA, United States) was used for identification. The fatty acid contents were quantified by normalization according to AOCS Method Ce 1f-96 (29).

### 2.3 Phytosterols analysis

The phytosterols present in the oils were determined by the method of Maguire and others (30). Briefly, the oils were saponified by refluxing in methanolic potassium hydroxide. Cholestane (Sigma Aldrich) was added as an internal standard. The solutions were extracted into ethyl ether. The extracts were washed with water to neutralize them and then evaporated under a nitrogen stream. The phytosterols were taken up in hexane and converted to their trimethylsilyl esters using Regisil® BSTFA (Regis Technologies Inc., Morton Grove, IL, United States). The extracts were analyzed with a Perkin Elmer Autosampler XL gas chromatograph (Shelton, CT, United States) using a flame ionization detector at 305 °C. A standard solution of phytosterols (Matreya) was treated as the oil extracts and run with the samples for identification and quantification. The column was an RTX-5 (30 m length, 0.25 mm internal diameter, 0.25-micron film, Restek Corp., Bellefonte, PA, United States). The temperature program was 100 °C held for 0.2 min, then increased at 10 °C/min to 305 °C and held for 15 min. The injector was split less at 235 °C. The carrier gas was helium at 2.1 mL/min.

### 2.4 Tocopherol analysis

The alpha (*α*-), Beta (*β*-), gamma (*γ*-) and delta (*δ*-) forms of tocopherols were determined in the oils as previously described (31). In brief, weighed quantities of the oils were diluted into the mobile phase (1% isopropanol in hexane). The solutions were injected onto an Agilent Model 1100 High Pressure Liquid Chromatograph. The column was a Luna Silica (250 mm length, 4.6 mm interior diameter, 5-micron packing, Phenomenex Corp.) heated to 25 °C. The flow rate was 1.2 mL/min. Detection was by UV at 294 nm. Standard curves were prepared from 1 μg/mL to 1,000 μg/mL using authentic standard purchased from Sigma Aldrich for identification and quantification.

## 3 Results and discussion

### 3.1 Fatty acids

Since vegetable oils are composed largely of triglycerides (98%), the fatty acids present will have the greatest effect on the characteristics of the oils (4). Oils are distinguished from fats in that the fatty acid present are mostly unsaturated whereas in fats, the majority of the fatty acids are saturated. Saturated fatty acids are essentially linear molecules. The unsaturated fatty acids are unable to pack tightly and thus oils remain liquid at room temperature and in some cases lower (7).

As seed oils, the fatty acids present at the highest levels were the unsaturated ones, allowing the oils to be free flowing at room temperatures (Table 1). The pecan and especially the sunflower oils had the highest levels of monounsaturated fatty acids. Monounsaturated fatty acids have been extensively studied for their health benefits, most notably in olive oil (32, 33). They have been associated with lower blood levels of triglycerides (TG) and higher levels of high-density lipoprotein cholesterol (HDLC), the so called “good cholesterol” (34). Olive oil is reported to contain 69% monounsaturated fatty acids (17). The pecan oil would be comparable to olive oil in monounsaturated fatty acid content and the sunflower an even higher source of this healthy lipid (35). The okra oil and the pecan oil contained significant levels of an omega-3 fatty acid (alpha linoleic). This type of fatty acid has been associated with a range of health benefits such as neurological function, blood pressure control, glucose metabolism, inflammatory response, and blood cell development (36).

**Table 1:** Fatty acid composition of cold pressed oils.

Fatty acid composition (%)	Benne	Okra	Peanut	Pecan	Pumpkin	Sunflower
Unsaturated fatty acid						
Palmitoleic acid (C16:1)	0.12	0.38	0.08	0.07	0.11	0.18
Oleic acid (C18:1 omega-9)	40.52	29.57	53.17	63.95	36.96	89.24
Cis linoleic acid (C18:2 omega-6)	43.14	31.33	26.30	25.86	44.01	1.89
Alpha linolenic acid (C18:3 omega-3)	0.25	1.24	0.11	1.17	0.15	0.04
Eicosenoic acid (C20:1 omega-9)	0.17	0.07	1.17	0.29	0.09	0.31
Erucic acid (C22:1 omega-9)	ND	0.27	0.08	ND	0.09	ND
Total monounsaturated fatty acids	40.81	30.29	54.50	64.31	37.25	89.73
Total polyunsaturated fatty acids	43.33	32.57	26.41	27.03	44.16	1.93
Saturated fatty acid						
Myristic acid (C14:0)	0.01	0.28	0.01	0.05	0.11	0.05
Palmitic acid (C16:0)	9.74	29.24	9.38	5.97	11.59	4.52
Heptadecanoic acid (C17:0)	0.04	0.11	0.10	0.06	0.09	0.03
Stearic acid (C18:0)	5.18	4.85	3.07	2.36	5.89	2.38
Arachidic acid (C20:0)	0.61	0.58	1.51	0.11	0.43	0.25
Behenic acid (C22:0)	0.10	0.28	3.22	0.01	0.11	0.82
Lignoceric acid (C24:0)	0.05	0.12	1.54	ND	0.02	0.27
Total saturated fatty acids	15.73	35.46	18.83	8.56	18.24	8.32

ND, None Detected.

As seeds develop, fatty acids are formed from acetyl-CoA by the addition of two carbon units inside the plastids of the cells (37). In simplest terms, the saturated fatty acids are then desaturated by enzymatic activity to produce the mono and then the polyunsaturated fatty acids. Oils from seeds are the best sources of polyunsaturated fatty acids which have been studied extensively for positive health effects, most notably for cardiovascular health and reduction of Type 2 Diabetes risk and improved insulin sensitivity (38). Several of the oils discussed here have been the topic of clinical studies and their positive health effects reported. These include okra (39), peanut (40), pecan (41), pumpkin (42), and especially sunflower (43). A current and very extensive review of the published studies emphasized the beneficial health effects of consumption of unsaturated fatty acids over saturated ones (44). Polyunsaturated fatty acids are more easily oxidized which produces off flavors due to rancidity and more importantly compounds such as lipid peroxides that are harmful to human health due to their role in increased risk of atherosclerosis and certain cancers as a result of inflammation (45). Refined oils contain synthetic antioxidants to prevent oxidation, but cold pressed oils do not. It is important to store cold pressed oils away from light and heat to avoid oxidation. They are also usually not recommended for high heat cooking such as deep frying as well. Certain oils such as the sunflower oil with the low polyunsaturated levels could be used for more moderate cooking methods such as sauteing.

Of the oils reported here, the okra oil contained the highest levels of saturated fatty acids followed by the pumpkin seed. While this may indicate consumption of these oils is less healthy than the others in this study, cold pressing reserves other compounds that may offset this as discussed in other sections following.

Although not listed in Table 1, hexacosanoic acid (C 26:0) was found only in the peanut oil here and has been reported in the literature (46). This and other very long chain fatty acid such as erucic acid (C 22:1) has been associated in theory in the oxidized form with symptoms of adrenoleukodystrophy such as progressive myelination and adrenal cortex insufficiency (47). However, when peanut oil was used to study the feeding effects of these very long chain fatty acids no influence on their levels in serum were seen (48). Knowing these compounds could be found in peanut and other cold pressed seed oils provides sources for future studies.

### 3.2 Phospholipids

Phospholipids were not measured for this work, but some information is available in the literature for oil from some of the seeds being discussed here. Including this information in the discussion contributes to the topic of lipid content of cold pressed oils. Table 2 lists some literature values for the oils evaluated for this report. The highest levels were found in the okra and the pumpkin seed and the lowest in the sunflower seed oil.

**Table 2:** Phospholipid composition of cold pressed oils (literature values).

Phospholipid (mcg/g oil)	Benne	Okra (69)	Peanut (70)	Pecan (71)	Pumpkin (72)	Sunflower (73)
PC	No data	No data	664	1,072	2,780	17–291
PE	No data	No data	113	1,013	674	104–162
PG	No data	No data	250	610	45	NR
PI	No data	No data	1,500	NR	693	109–189
PS	No data	No data	NR	116	68	NR
Total Phospholipids	No data	5,200–5,700	2,527	2,811	4,260	230–642

NR, not reported. PC, phosphatidylcholine; PE, phosphatidylethanolamine; PG, phosphatidylglycerol; PI, phosphatidylinositol; PS, phosphatidylserine.

One of the principal effects of the refining of vegetable oils is the removal of the phospholipids in the degumming step. Water and citric acid are added to the oil to cause the phospholipids to become too polar to remain solubilized in the oil (49). Phospholipids are removed to increase the oil yield and improve oil quality as phospholipids are easily oxidized and cause darker colors. Since phospholipids are excellent emulsifiers, they are recovered from the degumming process commercially to use as food and pharmaceutical ingredients (50, 51).

**Figure 1:**
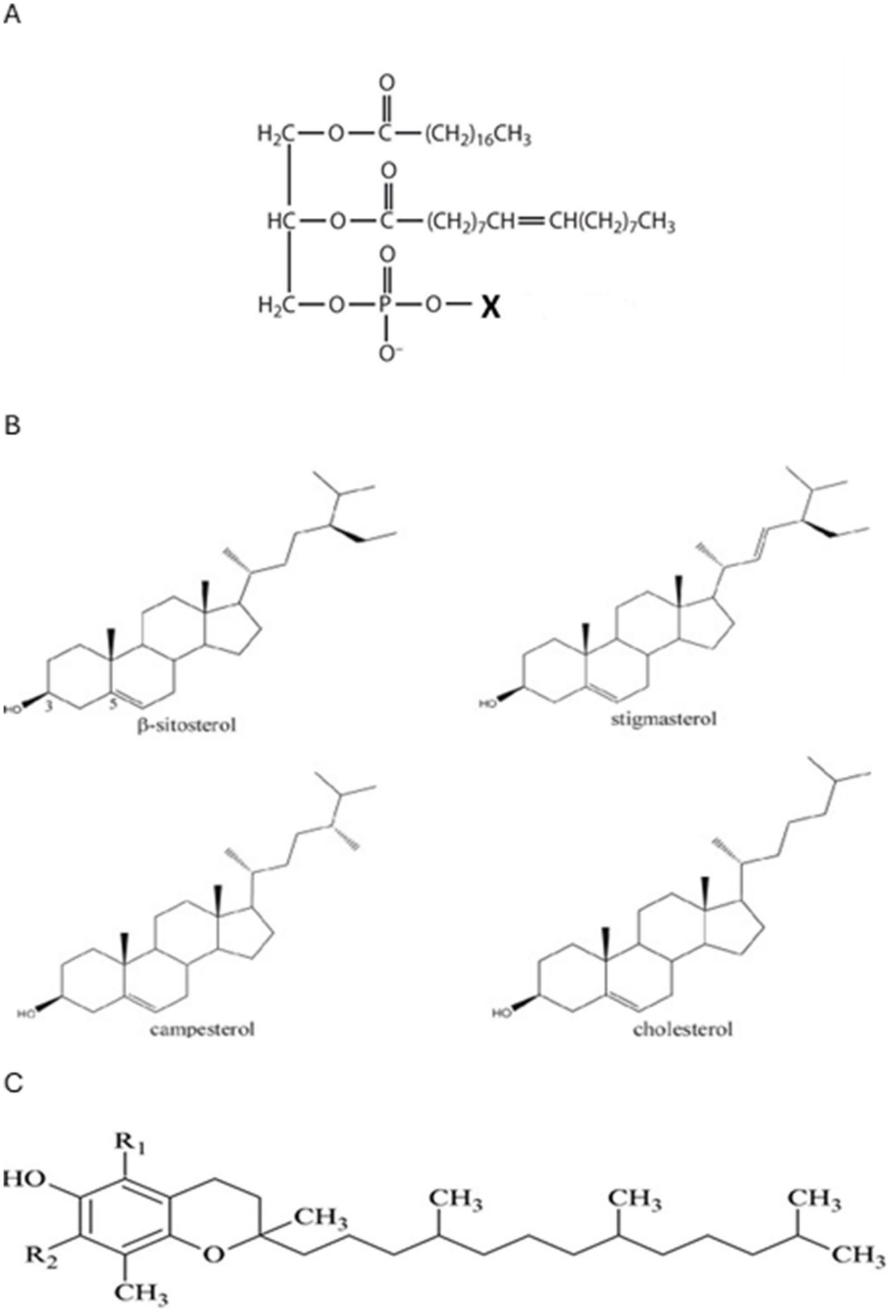
**(A)** Basic structure of a phospholipid molecule. The different hydrophilic groups that define the various phospholipids are bound at the site marked X. **(B)** Structures of the major plant sterols and cholesterol. **(C)** General structure of tocopherols found in seed oils. In Alpha, R_1_ and R_2_ are methyl groups (CH_3_). In Beta, R_1_ is a methyl group (CH_3_) and R_2_ is a hydrogen atom (H). In Gamma, R_1_ is a hydrogen atom (H) and R_2_ is a methyl group (CH_3_). In Delta, R_1_ and R_2_ are both hydrogen atoms (H).

Phospholipids are found in the membranes of cells and play a structural role. Since one end of the molecule consists of the phosphate group attached to a glycerol, it is hydrophilic and faces out to the cell fluid. The so-called tail end faces inward and is made up of 2 fatty acids (52). They have effects on the proteins that are embedded in the cell membrane and serve as messengers, as well as influence the secondary messengers by the content of their fatty acids (53). The major phospholipids found in foods are phosphatidylcholine (PC), phosphatidylethanolamine (PE), phosphatidylglycerol (PG), phosphatidylinositol (PI), and phosphatidylserine (PS). They all have similar structures with varying groups attached to the phosphate group at the head of the molecule (Figure 1A).

For human nutrition, there is no requirement for phospholipid intake, and the actual scientific evidence is lacking for their positive effects on human health (54). A wide range of studies exist to describe how they contribute to maintaining the antioxidants such as phenolics and tocopherols in oil, provide stability to the oil and may be antioxidants themselves (55). Using the isolated compounds, studies have been done to determine their effectiveness in as anti-inflammatories, tumor inhibition, blood lipid regulation, neurological development, liver disease treatment, and maintaining immunological function (53).

### 3.3 Phytosterols

Phytosterols or plant sterols serve as structural components to add rigidity to the cell walls of membranes in plants, whereas cholesterol performs the same function in animal cells (56). They are a group of secondary alcohols containing 27 to 29 carbon atoms in four connecting ring structures (Figure 1B).

The differences between the structures are the side chains. Campesterol contains an additional methyl group. Stigmasterol and *β*-sitosterol contain an ethyl group and stigmasterol has a double bond in the middle of the side chain. The phytosterols are present in plant oils as free sterols and esterified to fatty acids through the hydroxy group on the first ring. Plant sterols molecules are similar enough to cholesterol so that they are able to compete for the absorption sites within the human intestine but are not as easily absorbed. In doing so, they also prevent cholesterol absorption into the blood (57). They are additionally cholesterol lowering by increasing the excretion of cholesterol from the body via bile (58). Commercial refining of vegetable oil includes a treatment with absorbent clay or similar materials to remove contaminants that color the oil. This treatment results in the removal of some of the phytosterols as well, especially the esterified ones (59). Additional processes steps especially the deodorization, remove phytosterols (60). Cold pressed oils will retain all of their phytosterols as there is no additional treatment after the oil is extruded. The phytosterols contents determined in this study are reported in Table 3. The most abundant was *β*-sitosterol in all the oils tested. The okra seed oil had the lowest levels overall while the benne and the sunflower seeds had the highest. The pumpkin seed was richest in Campesterol, and the sunflower seed was highest in Stigmasterol.

**Table 3:** Phytosterol and tocopherol composition of cold pressed oils.

	Benne	Okra	Peanut	Pecan	Pumpkin	Sunflower
Phytosterol (mg/100 g)						
Campesterol	67.46	9.83	22.11	3.96	83.86	32.79
Stigmasterol	16.26	4.93	18.62	1.57	15.08	38.84
Β-sitosterol	232.96	22.13	100.65	67.11	68.78	251.81
Total Sterols	316.67	36.89	141.38	72.64	167.82	322.44
Tocopherol (mcg/g oil)						
Alpha (α-)	3,601	14.5	208	14.5	20.6	590
Beta (β-)	7,800	2.9	11.2	9.2	2.0	19.2
Gamma (γ-)	359	375	153	281	809	ND
Delta (δ-)	17.3	ND	14.6	6.3	19.9	2.4
Total Tocopherols	11,778	645	387	311	851	851

ND, None Detected.

Although not listed in the table, the oils were analyzed for Brassicasterol, but this was only found in the okra seed oil at 4.35 mg/100 g. Brassicasterol is typically found in cruciferous vegetables such as broccoli and cabbage, but also in rapeseed or canola oil (61). This is interesting since okra is not cruciferous. Brassicasterol is very similar to sigmasterol in that it has a double bond on the side chain, but has only a single methyl group attached to the end of the side chain rather than an ethyl group. The cold pressed oils could be considered to be healthier choices than refined vegetable oils with significant levels of the phytosterols being retained, especially in the sunflower oil.

### 3.4 Tocopherols

Tocopherols are a group of lipid soluble compounds that serve as natural antioxidants for plant cell membranes and organs (62). Most plant seed oils are sources of tocopherols also referred to as Vitamin E. They all have the same general structure as seen in Figure 1C. The different forms are defined by the moiety attached at the positions designated by R_1_ and R_2_ on the aromatic ring. The ring structure causes the molecule to absorb light in the visible spectrum, resulting in tocopherols appearing yellow in color.

Peanut oil is reported to be a good source of Vitamin E in the form of *α*-tocopherol (63). Whereas tree nuts such as almonds and hazel nuts have high levels of α-tocopherols and low levels of *γ*-tocopherols (64), edible seeds such as pumpkin and sesame have high levels of γ-tocopherol and low levels of *α*-tocopherol which have been found to be related to the levels of polyunsaturated fatty acids present in the oil (65). Peanuts seem to be rather unique in having similar significant levels or each type.

In terms of value to human nutrition, the Vitamin E content of cold pressed oils is an important parameter. Humans require Vitamin E to protect cell membranes from oxidation (66). Membranes contain polyunsaturated fatty acids, especially in the mitochondria and the endoplasmic reticulum which are easily oxidized if not protected. Other oxidation can occur from free radical oxygen attack which Vitamin E can mitigate. As the most biologically active form, only α-tocopherol is reported as Vitamin E on food labels. Gamma tocopherol has only about 10% of the activity as the alpha form (62).

Several steps, especially decolorization and deodorization, in the refining of seed oils are known to remove tocopherols (60). Cold pressing preserves the tocopherols. The values found for the tocopherols in the oils in this study are reported in Table 3.

The Benne seed oil was found to contain the highest levels with almost twice that of the other oils. Most unusually, the Beta form was the most prevalent whereas in most vegetable oils, the levels of this type are usually negligible. In the biosynthesis pathway for tocopherol formation in seeds, a precursor molecule MPBQ (2-methyl-6-phytyl-1,4-benzoquinol) forms the delta form of tocopherol or is methylated to DMPBQ (2,3-dimethyl-6-phytyl-1,4-benzoquinol) which can then go on to form the alpha form (67). The Beta is synthesized from the delta form which indicates the genetic response is extremely strong in the Benne seed. As the gamma form is converted to the alpha form, the okra and pumpkin seeds have similar activity. Sunflower seed oil is produced from a wide range of cultivars. Values for the gamma form for sunflower cover a wide range from none to over 600 mcg/g, which has resulted in studies of the genes controlling their formation (68). The report here of no *γ*-tocopherol found agrees with previous reports. Peanut oil appears to be unique in that equal amounts of alpha – and gamma-tocopherol are found. From a nutritional perspective, preservation of tocopherols, especially alpha tocopherol increases the value of cold pressed oils over that of refined ones.

## 4 Conclusion

The cold pressing of oils without further refinement produces distinct products from industrial refined vegetable oils for food use. The process is less economically efficient due to the lower oil yields from the seed source. Other disadvantages include low smoke points which limit cooking applications requiring high heat such as deep frying. Cold pressed oils will also become cloudy at low temperatures due to presence of minor lipid components such as phospholipids. Viscosities may also be different which may affect their uses as food ingredients in products such as baked goods. The preservation of minor lipid components by cold pressing results in products that have higher nutritional qualities. Phytosterols and vitamin E are retained. The small volatile and semi-volatile compounds that provide distinct and pleasing flavors and aromas are also retained producing products that are prized for their sensory qualities which complement their culinary uses.

## Data Availability

The original contributions presented in the study are included in the article/supplementary material, further inquiries can be directed to the corresponding author.
